# A case report of avian influenza H7N9 killing a young doctor in Shanghai, China

**DOI:** 10.1186/s12879-015-0970-4

**Published:** 2015-06-23

**Authors:** Hao Pan, Xi Zhang, Jiayu Hu, Jian Chen, Qichao Pan, Zheng Teng, Yaxu Zheng, Shenghua Mao, Hong Zhang, Chwan-Chuen King, Fan Wu

**Affiliations:** Department of Infectious Disease Control and Prevention, Shanghai Municipal Center for Disease Control and Prevention, No 1380, West Zhongshan Road, Shanghai, 200336 China; Department of Infectious Disease Control and Prevention, Pudong District Center for Disease Control and Prevention, No 3039, ZhangYang Road, Shanghai, 200136 China; College of Public Health, National Taiwan University, Taipei, 100 Taiwan

**Keywords:** Avian influenza H7N9, Live-poultry market, Healthcare workers, Preventive measures, Public health policies

## Abstract

**Background:**

The novel avian influenza H7N9 virus has caused severe diseases in humans in eastern China since the spring of 2013. On January 18^th^ 2014, a doctor working in the emergency department of a hospital in Shanghai died of H7N9 virus infection. To understand possible reasons to explain this world’s first fatal H7N9 case of a health care worker (HCW), we summarize the clinical presentation, epidemiological investigations, laboratory results, and prevention and control policies and make important recommendations to hospital-related workers.

**Case presentation:**

The patient was a 31-year-old male Chinese surgeon who was obese and had a five-year history of hypertension and suspected diabetes. On January 11^th^ 2014, he showed symptoms of an influenza-like illness. Four days later, his illness rapidly progressed with bilateral pulmonary infiltration, hypoxia and lymphopenia. On January 17th, the case had a high fever, productive cough, chest tightness and shortness of breath, so that he was administered with oseltamivir, glucocorticoid, immunoglobulin, and broad-spectrum antibiotic therapy. The case died in the early morning of next day after invasive ventilation. He had no contact with poultry nor had he visited live-poultry markets (LPMs), where positive rates of H7N9 were 14.6 % and 18.5 %. Before his illness, he cared for three febrile patients and had indirect contact with one severe pneumonia patient. Follow-up with 35 close contacts identified two HCWs who had worked also in emergency department but had not worn masks were anti-H7N9-positive. Viral sequence identity percentages between the patient and two LPM-H7N9 isolates were fewer than between the patient and another human case in shanghai in January of 2014.

**Conclusions:**

Important reasons for the patient’s death might include late treatment with oseltamivir, and the infected H7N9 virus carrying both mammalian-adapted signature (HA-Q226L) and aerosol transmissibility (PB2-D701N). The LPM he passed every day was an unlikely source of his infection, but a contaminated environment, or an unidentified mild/asymptomatic H7N9 carrier were more probable. We advocate rigorous standard operating procedures for infection control practices in hospital settings and evaluations thereafter.

## Background

The first human avian influenza H7N9 case was reported in Shanghai in February 2013 [1]. By the end of 2013, Shanghai had 33 laboratory-confirmed human H7N9 cases, with a higher case fatality rate (CFR) than observed nationally [54.6 % (18/33) versus 32.6 %, (47/144)]. As of September 27^th^ 2014, Shanghai had an additional 8 cases with 7 fatalities [CFR in 2014: 87.5 % (7/8) versus 42.2 %, (125/296)]. Two family clusters were noted in Shanghai, indicating limited person-to-person transmission [2]. On January 18^th^ 2014, the first HCW succumbed to H7N9. In this report, we summarize the clinical presentation, epidemiological investigations, laboratory results, and prevention and control policies and make recommendations.

## Case presentation

The case under consideration in this article is a 31-year-old male surgeon working in the emergency department (ED) of a Pudong hospital in Shanghai (SH-PDH), China. He was obese (BMI: 29.39, ≥28 in China [3]), with a five-year history of hypertension and suspected diabetes, and was a non-smoker. There was no history of previous drug or food allergies or blood transfusions.

### Clinical history

**Fig. 1:**
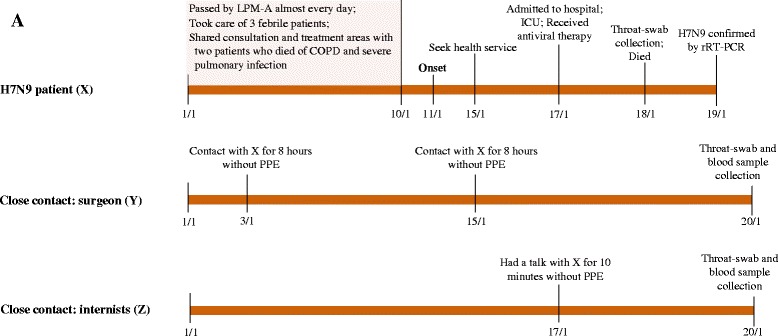
Timeline of the H7N9 patient’s illness, treatment, death and his close contacts. PPE: personal protective equipment

**Fig. 2:**
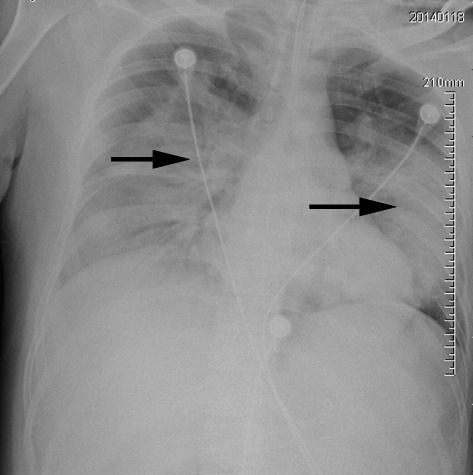
Representative radiographic findings of the laboratory-confirmed 31-year-old Shanghai surgeon infected with H7N9 influenza. Chest radiograph of this patient was taken at 7 days after onset of symptoms, showing bilateral pulmonary infiltrates of airspace consolidation and severe consolidation in the left lobe

On January 11^th^ 2014, the patient showed symptoms of an influenza-like illness (ILI) (fever, cough, sore throat, dizziness, headache and myalgia) and self-treated with Analginum (Fig. 1). Four days later, the surgeon sought medical care and took mezlocillin only. From January 11^th^ to January 16^th^, he continued working (~8 hours a day) at the hospital until he developed dyspnea. He was not treated with oseltamivir prior to his admission into SH-PDH intensive-care-unit (ICU) on January 17^th^. His illness rapidly progressed with bilateral pulmonary infiltration, hypoxia and lymphopenia. Oxygen therapy and mechanical ventilation were started. Additionally, oseltamivir (75 mg orally and 150 mg intra-gastrically), glucocorticoid, immunoglobulin and broad-spectrum antibiotics therapy (imipenem and vancomycin, 1 g intravenously every 12 hours) were administered. At 8:00 AM on January 17^th^, he had a fever (39 °C), productive cough, chest tightness and shortness of breath. The white blood cell count was 6.20 × 10^9^/L with 83.4 % neutrophils and 14.5 % lymphocytes (Table 1). A computed tomography chest scan showed consolidation in both lungs (Fig. 2). At 8:47 AM, the patient was given non-invasive ventilation but he continued to suffer from hypoxaemia. As his condition worsened, he was started on invasive ventilation with positive end-expiratory pressure at 11:28 AM. The patient died of acute respiratory distress syndrome, severe pneumonia, and type I respiratory failure at 4:59 AM on January 18^th^.

**Table 1:** Clinical characteristics of the 31-year-old-case

Characteristics	Patient (at 3:00 AM on January 17^th^)	Normal value
**Clinical symptoms/signs**		
Fever	39.0 °C	-
Cough	Yes	-
Expectoration	Yes	-
Cough with blood tinged sputum	No	-
Sore throat	Yes	-
Dizziness	Yes	-
Headache	Yes	-
Myalgia	Yes	-
Shortness of breath	Yes	-
Dyspnea	No (at 3:00 AM) but Yes (at 8:00 AM) on January 17^th^	-
Chest pain	No	-
Abdominal pain	No	-
Diarrhea	No	-
Nausea	No	-
Vomiting	No	-
Skin ecchymosis	No	-
Coma	Yes	-
**Blood cell count**		
White blood cell	6.2 × 10^9^/L	3.5-9.5 x10^9^ cells/L
Neutrophils	83.4 %	50.0 %-70.0 %
Lymphocytes	14.5 %	20.0 %-40.0 %
**Blood gas analysis**		
PH	7.4	7.4-7.5
PO_2_	42.9 mmHg	83.0-108.0 mmHg
PCO_2_	31.7 mmHg	22.0-29.0 mmHg
SPO_2_	83.7 %	95.0-98.0 %
**Chest findings**		
Chest computed tomography on 17 January	Consolidation	-
**Complications**		
Septic shock	No	-
Respiratory failure	Yes	-
Acute respiratory distress syndrome	Yes	-
Acute renal damage	No	-
Encephalopathy	No	-
Multiple organ failure	No	-
Diffuse intravascular coagulation	No	-
Secondary infections	No	-
**Treatment**		
Oxygen therapy	Yes	-
Extracorporeal membrane oxygenation	No	-
Continuous renal replacement therapy	No	-
Antibiotic therapy	Mezlocillin, imipenem and vancomycin	-
Antiviral agent	Oseltamivir	-
Glucocorticoid therapy	Methylprednisolone	-
Intravenous immunoglobulin therapy	Yes	-
Mechanical ventilation	Positive end expiratory pressure	-

### Field epidemiology investigation

Retrospective investigation showed that X lived with his six family members (including his 7-month-pregnant wife) in a 112-m^2^-sized house without birds or other animals. Although he had not visited LPMs, he usually rode a bicycle to work and passed by the LPM -A where bird feces were spread on the road near the market gate. His mother-in-law did grocery shopping at the LPM-B which is 2.5 miles from X’s home almost every day. One of his neighbors owned about 14 pigeons. On January 19^th^-20^th^, 2014, six pigeon-related samples (2 poultry feces, 1 throat swab, 1 drinking water, 1 polluted water, and 1 swab of cage) from X’s neighbor and 68 LPM-associated samples collected from LPM-related samples [including throat/anal swabs, and feces of poultry and environmental specimens (chicken cages, cutting boards, water from washing poultry flowing on the floor)] were tested by real-time reverse-transcriptase-polymerase-chain-reaction (rRT-PCR) [4]. The results showed that the positive rates of H7N9 were 14.6 % (6/41), 18.5 % (5/27), and 0 % (0/6) from LPM-B-, LPM-A- and pigeon-related specimens, respectively. Ten days before the onset of X’s illness, all six family members were asymptomatic and no guests had visited.

**Fig. 3:**
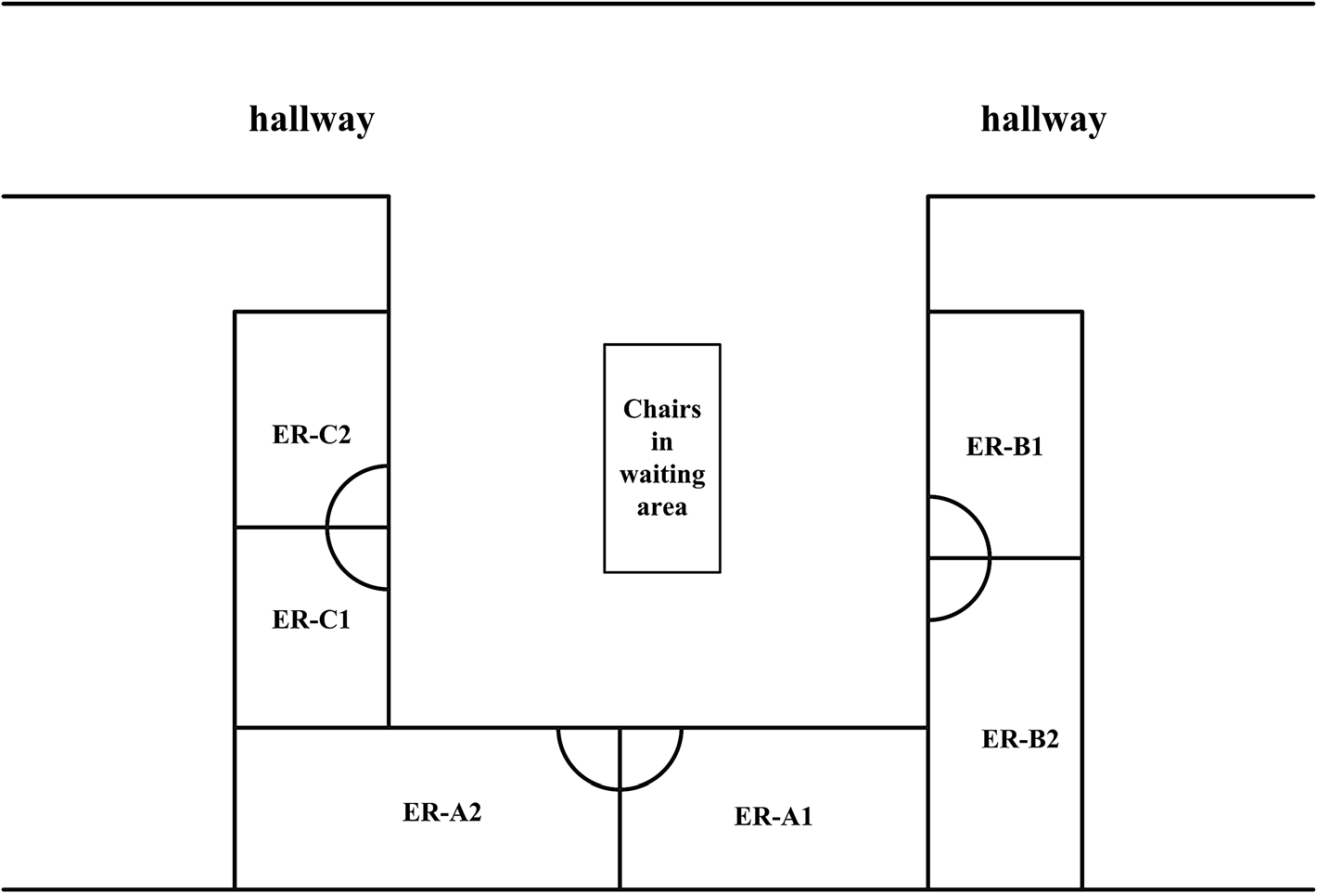
Spatial distribution of the six consultation rooms in the emergency department of the Pudong Hospital. ””: door of consultation room; ER: emergency room; H7N9-(+) patient X and his close contact Y worked in ER-A1. In addition, X and another close contact Z worked in ER-C1. The COPD-1 visited ER-C on January 7 and SP-1 visited ER-C on January 5. ER-B1 is used for emergency handling and case management of the patients from ER-A and ER-C, shared by the two departments of Surgery and Internal Medicine. ER-B2 was used for cleaning trauma by ED-surgeons. Surgeons in ER-A generally did not wear oral masks

At the hospital (SH-PDH), the ED is a 400-m^2^ U-shaped emergency room, including six 10-m^2^-sized neighboring rooms, where neither windows nor systematic regular ventilation system were installed. The ER-A and ER-C (Fig. 3) consultation rooms were used by the Departments of Surgery and Internal Medicine, respectively. In the 10 days before disease onset, X took care of 187 surgical patients in ER-A1. Three of X’s patients who developed febrile illness and recovered completely were migrants lost to follow-up. Further investigations revealed that two other patients who had sought medical care in ER-C during January 1^st^-10^th^ 2014 had also died. One was a ninety-seven-year-old man with chronic obstructive pulmonary disease (COPD-1) who arrived at ER-C on January 7^th^, talked with an ED-internist (Dr.-A1), was immediately hospitalized [cared for by 3 doctors (Drs.-A2, A3, and A4) and one nurse (N-A1)], and died the next day. The other was an eighty-eight-year-old woman with severe pneumonia (SP-1) who went to ER-C on January 5^th^, consulted an ED-internist Dr.-B1, was subsequently hospitalized (cared for by Drs.-B2, B3, and B4, and N-B1), and died on January 8^th^ from an infection that was not identified. Specimens from 10 persons [COPD-1, SP-1, 8 HCWs except Dr.-A1 andDr.-B1] were not available for H7N9 tests. Follow-up of all 10 HCWs who had direct contact with COPD-1 and SP-1 showed that none of them became ill (no fever/cough/pneumonia) between January 10^th^ 2014 and January 20^th^ 2014. In addition, both the throat and blood specimens collected on January 20^th^ from Dr.-A1 and Dr.-B1 in ER-C, who shared the same space or air with X in ER-A1, were H7N9-negative.

### Identification and Tracing of close contacts

In our investigation of human-to-human transmission, we defined close contacts of X as those who had direct contact but without taking any personal protective equipment (PPE) between January 11^th^ and January 18^th^. In total, 35 close contacts of X were identified for daily monitoring, including 9 family members (3 visited him at the hospital but lived separately) and 26 HCWs [11, 10, 2, and 3 from ED, ICU, radiology, and supporting staff, respectively]. None had respiratory or other H7N9-related symptoms within 10 days of their last exposure to X. On January 20th, we collected throat swabs for rRT-PCR, and all samples were H7N9-negative. Since serological surveillance provides more information on total infection, we then tested serum samples collected from all 35 close contacts by haemagglutination inhibition (HAI) assay [5], using influenza A/Anhui/1/2013 (H7N9) virus as a viral antigen and horse red blood cells. Furthermore, the two anti-H7N9-positive HCWs were investigated in detail. The HCW with a serotiter of 1:40 against the H7N9 virus was a 37-year-old male ED surgeon (Y), and the other one with a serotiter of 1:20 was a 30-year-old male ED internist (Z). These three HCWs (X, Y, and Z) did not care for the same patient from January 1^st^ to January 17^th^. They had never worked in the ICU and did not have any overlapping encounters with patients of COPD-1 and SP-1. X and Y had worked together but had not worn masks in ER-A1 for two days (8 hours/day) on January 3^rd^ and 15^th^ January (4 days after the onset of X’s illness). In addition, X talked with Z in ER-B1 (for emergency handling of patients) for 10 minutes without their masks at 3:00 AM on January 17^th^ before being admitted to the ICU. Sixteen environmental samples were collected from ER-A1 (the surface of telephone receiver, printer, work table, and medical waste), ER-B2 (rails of hospital beds, oxygen humidifier, sputum aspirator surface, ECG monitor wall, outer surface of IV stand), and ICU (ECG monitor wall, surface of blood pressure meter, outer surface of sputum aspirator, Infusion pump surface, stethoscope surface and rails of hospital beds). They were H7N9-negative by rRT-PCR.

### Virology and associated molecular investigations

On January 18^th^ 2014, the throat swab of X collected on the day of death was sent to Shanghai Municipal Center for Disease Control and Prevention, and confirmed as H7N9 by rRT-PCR on January 19^th^. Phylogenetic analysis for all eight H7N9 viral genomic segments was performed by both neighbour-joining (NJ) and maximum-likelihood (ML) methods with MEGA version 5.10. The reliability of the unrooted tree was assessed by bootstrap with 1000 replications. Bootstrap values greater than 60 % are shown for selected nodes. All the nucleotide sequences of the eight viral gene segments of X’s H7N9 virus (A/Shanghai/PD-02/2014, PD-2) are available at GenBank with the following accession numbers: KJ549801 (PB2), KJ549802 (PB1), KJ549803 (PA), KJ195797 (HA), KJ195798 (NP), KJ195799 (NA), KJ195800 (M), KJ195801 (NS).

The eight viral genes shared between X’s H7N9 virus (A/Shanghai/PD-02/2014, PD-2) and the avian-origin H7N9 virus isolates from the two LPMs in this study (A/Chicken/Shanghai/PD-CN-02/2014, CN-2 and A/Environment/Shanghai/PD-JZ-01/2014, JZ-1) revealed nucleic acid sequence identity percentages ranging from 97.8 % to 99.5 % (Table 2), in which the percentages of the PA gene ranked lowest (97.9 %, 97.8 %). However, the overall viral sequence identity was higher (ranging 98.1 % ~ 99.7 %) between the two Shanghai human cases, PD-2 and PD-1 (A/Shanghai/PD-01/2014, PD-1), while PD-1 was another non-epidemiologically linked H7N9 human case that was also confirmed in January in SH-PD. Similarly, viral sequence patterns for all the eight individual genes were also closer between PD-2 and PD-1 than those between PD-2 and CN-2 or JZ-1. GenBank accession numbers of PD-2, JZ-1, and CN-2 are as follows: PB2 (KJ549799, KJ549791, KJ549783), PB1 (KJ195791, KJ549792, KJ549784), PA (KJ549800, KJ549793, KJ549785), HA (KJ195792, KJ549794, KJ549786), NP (KJ195793, KJ549795, KJ549787), NA (KJ195794, KJ549796, KJ549788), M (KJ195795, KJ549797, KJ549789), NS (KJ195796, KJ549798, KJ549790).

**Table 2:** The nucleic acid identity percentages among four H7N9 isolates

Segment of H7N9	Identity with CN-2 (from LPM-A)	Identity with JZ-1 (from LPM-B )	Identity with PD-1	Identity with PD-2
PB2	97.9	98.9	99.1	reference
PB1	99.4	99.0	99.7	
PA	97.9	97.8	98.1	
HA	99.1	99.4	99.5	
NP	99.5	99.1	99.7	
NA	98.8	98.6	99.3	
M	98.3	98.4	98.4	
NS	99.2	99.2	99.4	

PD-2: the surgeon’s H7N9 isolate; PD-1: another human case in the same district of Pudong as the PD-2 case; CN-2 and JZ-1: the two environmental H7N9 isolates obtained from the two live-bird markets. LPM-A is located at 200 meters from his hospital. LPM-B is the market (2.5 miles from his home) that the surgeon’s mother-in-law visited almost every day.

**Fig. 4:**
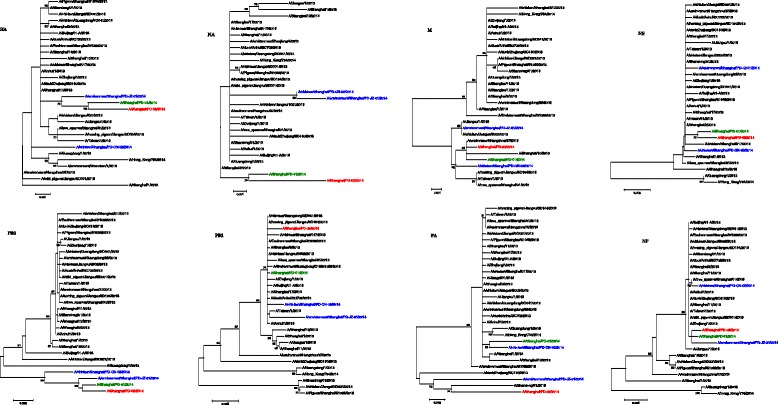
Phylogenetic relationships of the eight full-length genes of A/Shanghai/PD-02/2014. Horizontal distances are proportional to the genetic distance. Three colors represent the three different types of the sources of H7N9 viruses: (1) the A/Shanghai/PD-02/2014 (H7N9) virus was isolated from this young surgeon (X) is shown in red, (2) the A/Shanghai/PD-01/2014 (H7N9) virus was isolated from another PD patient in January without epidemiological linkage is shown in green, and (3) H7N9 viruses from the two LPMs are shown in blue. A/Chicken/Shanghai/PD-CN-02/2014 virus was isolated from the LPM-A near H7N9-(+) patient X’s hospital. A/Environment/ Shanghai/ PD-JZ-01/2014) was isolated from the LPM-B closer to H7N9-(+) patient X’s home

The phylogenetic tree topology was the same, using ML and NJ methods; hence the results of MJ tree are displayed in Fig. 4. The results showed that all the four genes (HA, NA, M and NS) of PD-2 clustered with PD-1 (i.e. the same sub-lineage) whereas the two LPM-derived H7N9 isolates belonged to another sub-lineage. HA, PA, PB2, and NP of JZ-1 are at the same branch as those of PD-2. The PD-2 had more amino acid changes from avian to human signatures, with markers of mammalian-host adaptation [1], such as HA-Q226L HA-G228S (H3 numbering), and PB2-E627K. In contrast, CN-2 and JZ-1 isolates kept one avian-associated molecular signature with no change at PB2-E627 among these three amino acids. For clinical concern, the PD-2 virus was still oseltamivir-sensitive (NA-E119 [6], R292 [5], and N294 [1]) but Amantadine-resistant (M2-S31N) [1, 7]. Most importantly, of these four SH-H7N9 viruses, only the PD-2 possessed PB2-D701N (aerosol transmissibility in ferrets) [8, 9], which has public health significance.

The study was approved by the Ethical Committee of the Shanghai Municipal Center for Disease Control and Prevention.

## Conclusions

We describe the world’s first case of a health care worker who died of H7N9 at the age of 31. This is much younger than the median age of 64 in 172 fatal H7N9 cases in China. Undoubtedly, the death of this young doctor could have several explanations. These include the fact that he suffered from hypertension, diabetes, and obesity. As of September 27^th^ 2014, these three underlying conditions were also present in 27.3 %, 11.1 %, and 10.7 % of 172 fatal H7N9 cases (131 with BMI data) in China, respectively. Obesity was also a risk factor contributing to the severity of the 2009 H1N1 influenza pandemic cases in China and other areas [10–12]. Perhaps more importantly, the patient was late to seek treatment (4 days after disease onset) [2] since he was unaware of his infection with H7N9. As a result, treatment with oseltamivir was not begun until 6 days after the onset of illness, a time when the drug would be ineffective [13]. Additionally, of critical importance, the patient was infected with a H7N9 virus that contained a mammalian-adapted signature (HA-Q226L), a mutation at PB2-D701N which facilitated its aerosol transmission as well as virulence markers (PB2-627 K and HA-G228S) [1, 14]. Taken together, along with the fact that the doctor had been working a seven-day shift with little rest since the onset of his disease, his lymphopenia indicating abnormal immunity [13, 15], and possibly unidentified accumulated exposures, these factors could explain the fatality at such a young age.

Identifying the source of infection is important to understand how the case may have acquired his H7N9 infection. The high positive rates of H7N9 at the two LPMs (14.6 % and 18.5 %) suggest that the H7N9 virus might be contaminating the markets and surrounding areas, providing potential for spread of the virus to the LPMs workers and/or shoppers. However, phylogenetic results did not indicate an LPM-A-oriented infection source; even X passed the entrance of LPM-A every day. Because four genes (HA, PA, PB2, NP) of JZ-1 clustered with PD-2, X’s infection might originate from LPM-B; but X wasn’t exposed directly to the environment and poultry from PLM-B. X’s mother in law might carry H7N9 virus to X’s home through the contaminated water in LPM-B. Finally, there is still some uncertainty about avian-origin since LPM-related specimens were taken after, rather than before, his death.

On the hospital side, environmental specimens were taken after thorough clean-up. Unfortunately, specimens from the three febrile patients cared for by X and the two fatal cases (COPD-1 and SP-1) were not available. Most importantly, the positive anti-H7N9 HCWs (Y and Z) from the serological surveillance demonstrated that contaminated environment-to-human or human-to-human transmission in a hospital setting is likely [16] if HCWs did not have appropriate PPE while caring for infected patients. The low HAI serotiter of Z might be due to a shorter exposure. None of the six family members of X (including his 7-month-pregnant wife) were infected, showing that the person-to-person transmissibility within this family setting was still limited. The source of infection was not identified. The LPM-A was an unlikely source, but a contaminated environment, or an unidentified mild/asymptomatic H7N9 carrier were more probable.

To minimize the health threat to citizens [17], the Shanghai Government announced the closure of LPMs from January 31^st^ to April 30^th^ 2014. There have been no H7N9 cases in Shanghai since February. Based on our experience, we recommend: (1) during epidemics of H7N9, those HCWs who might be exposed to H7N9 cases are advised to take oseltamivir or other available drugs against H7N9 viruses in advance; (2) implementing rigorous standard operating procedures for infection control practices, including PPE, traffic control [17], and evaluations thereafter; (3) enhancing surveillance by reporting of any HCWs who develop ILI; (4) avoiding possible cross-transmission among HCWs, patients, and visitors, particularly the immunocompromised and/or those with other co-morbidities [18, 19]; and (5) during H7N9 epidemics, H7N9 tests need to be performed on all severe cases of respiratory infection, severe pneumonia cases in particular, even these cases have no obvious epidemiologic exposures.

## Consent

All those discussed individuals provided written consents for the publication of this information and any accompanying images.

## Abbreviations

LPM: live-poultry market
HCW: healthcare worker
CFR: case fatality rate
ED: emergency department
SH-PDH: a Pudong hospital in Shanghai
ILI: influenza-like illness
ICU: intensive care unit
rRT-PCR: real time reverse-transcriptase-polymerase-chain-reaction
COPD: chronic obstructive pulmonary disease
SP: severe pneumonia
HAI: haemoagglutination inhibition
NJ: neighbour-joining
ML: maximum-likelihood
PD-1: A/Shanghai/PD-01/2014
PD-2: A/Shanghai/PD-02/2014
JZ-1: A/Environment/Shanghai/PD-JZ-01/2014
CN-2: A/Chicken/Shanghai/PD-CN-02/2014
